# Novel AI-derived muscle ultrasound biomarkers linking nutritional status and prognosis in cancer patients

**DOI:** 10.3389/fnut.2026.1961328

**Published:** 2026-10-14

**Authors:** Juan José López-Gómez, Lucía Estévez-Asensio, Paloma Pérez-López, Iván Rodríguez-Marín, Noelia Martín-Gómez, Olatz Izaola-Jauregui, Ángela Cebriá, Jaime González-Gutiérrez, Rebeca Jiménez-Sahagún, Puerto Bernoy-González, Emilia Gómez-Hoyos, Eduardo Jorge Godoy, Daniel A. De Luis-Román

**Affiliations:** 1 Servicio de Endocrinología y Nutrición, Hospital Clínico Universitario de Valladolid, Valladolid, Spain; 2 Centro de Investigación en Endocrinología y Nutrición, Facultad de Medicina, Universidad de Valladolid, Valladolid, Spain; 3 Health Research Institute of Valladolid (IBioVALL), Valladolid, Spain; 4 DAWAKO Medtech S.L., Parc Cientific de la Universitat de Valencia, Paterna, Valencia, Spain; 5 Técnicas Avanzadas de Desarrollo de Software Centrado en la Persona, Departamento de Informática, Universitat de Valencia, Burjassot, Valencia, Spain

**Keywords:** artificial intelligence, cancer, disease-related malnutrition, muscle ultrasonography, sarcopenia

## Abstract

**Introduction:**

Malnutrition and sarcopenia worsen prognosis in patients with cancer and require accessible diagnostic methods for assessing muscle quantity and quality. Artificial intelligence (AI)-assisted muscle ultrasound may improve the standardization and efficiency of body-composition assessment. This study evaluated the prognostic relevance of AI-assisted rectus femoris ultrasound biomarkers within a comprehensive nutritional assessment of patients with cancer.

**Methods:**

This prospective longitudinal observational cohort study included 389 patients with cancer, most of whom had tumors of digestive origin. Participants underwent longitudinal and transverse rectus femoris ultrasonography. Images were analyzed using PIIXMED™ MSK, which applies U-Net based anatomical segmentation followed by deterministic image-processing procedures. The evaluated variables included subcutaneous adipose tissue thickness (SAT), rectus femoris thickness (RFT) rectus femoris cross-sectional area (RFA), pennation angle, mean echo intensity, and the percentages of low- and medium-echogenicity pixels within the segmented region of interest, reported as the Muscle Index (Mi) and Fat Index (FATi), respectively. The muscle-area index was calculated as AreaMi = RFAxMi.

**Results:**

The 389 participants had a mean age of 70.6 (10.8) years, 58.9% were men, and 53.7% (*n* = 206) were classified as geriatric oncology patients (≥70 years). Malnutrition was present in 75.3% of participants, confirmed sarcopenia in 18.8% and overall mortality during follow-up was 26.5%. In univariate log-rank survival analysis, reduced muscle-area index (MiArea<1.36 cm^2^; *p* = 0.014) and low muscle quality (Mi < 46%; *p* = 0.019) were the strongest univariate predictors of significantly shorter survival. In multivariate Cox proportional Hazards regression models adjusted for age, sex, digestive tumor location, and metastatic disease, larger rectus femoris area (RFA: HR = 0.77. 95%CI: 0.62–0.95; *p* < 0.05) and thickness (RFT: HR = 0.43, 95% CI: 0.19–0.99; *p* < 0.05) remained independent protective factors against mortality. Conversely, reduced muscle index (Mi < 47%: HR = 1.60, 95%CI: 1.05–2.06; *p* < 0.05) and the presence of myosteatosis (HR = 2.35, 95%CI: 1.19–4.62; *p* = 0.01) independently predicted an increased risk of mortality.

**Conclusion:**

In cancer patients, AI-assisted rectus femoris ultrasound variables effectively predict mortality. Greater muscle area and higher muscle index (Mi > 47%) indicate a favorable prognosis. Early ultrasound assessment detects structural alterations before overt deterioration, providing a crucial window for targeted nutritional interventions to preserve muscle integrity and prevent complications.

## 1 Introduction

Malnutrition represents a highly prevalent comorbidity in the oncological population, affecting approximately 41% of patients globally based on GLIM criteria (1). Concurrently, sarcopenia, which frequently manifests secondary to disease-related malnutrition, has a reported prevalence of 35.3% (2). The incidence of both conditions is significantly elevated in older adults and in the context of advanced-stage disease, and their concurrent presentation is common and associated with synergistic adverse outcomes. Malnutrition is significantly associated with increased mortality (HR = 2.04) and higher incidence of postoperative complications (HR = 1.90). Independently, sarcopenia correlates with elevated mortality (HR = 1.43), greater chemotherapy toxicity (RR = 1.44), and poorer clinical outcomes following immunotherapy (RR = 1.58).

Given the high prevalence of malnutrition among oncology patients and its adverse consequences, European Society of Clinical Nutritional and Metabolism (ESPEN) guidelines recommend early nutritional screening to detect both established malnutrition and patients at risk of developing it (3). Despite the availability of various diagnostic tools, the objective assessment of malnutrition is hindered by intrinsic limitations such as interobserver variability, cost, and lack of validation (4).

Traditional metrics like body mass index (BMI) and current screening tests are inadequate for accurately assessing body composition and adiposity, often yielding false negatives (5). While dual x-ray absorptiometry (DXA), magnetic resonance imaging (MRI), and computed tomography (CT) serve as gold standards, their routine clinical use is limited by high costs, restricted availability, and repeated radiation exposure (6). Comprehensive nutritional assessment requires fast, accessible, and non-invasive point-of-care methods. Bioelectrical impedance analysis (BIA) and muscle ultrasound are key tools: BIA offers prognostic biomarkers of cellular health, while ultrasound provides portable, radiation-free evaluation of muscle quantity and quality. Although further standardization is needed (7).

Ultrasound is an exceptionally useful point-of-care tool for evaluating both muscle quantity and quality. This technique enables the visualization of muscle architecture, the quantification of muscle thickness, and the detection of qualitative alterations such as fat infiltration (myosteatosis) through increased echogenicity, a central pathological process in the development of cancer cachexia (8). Although the quadriceps femoris is inherently susceptible to early atrophy, this structural deterioration is drastically accelerated in oncological patients due to the systemic pro-inflammatory state and antineoplastic treatments (9). In this context, ultrasound-derived measurements of low muscle mass in the lower extremities show a strong correlation with clinical frailty (10). Specifically in patients with cancer, a decrease in the cross-sectional area and thickness of the rectus femoris muscle has emerged as an adverse prognostic biomarker, directly associated with poorer therapeutic responses and increased mortality (11).

Although muscle ultrasound is a valuable diagnostic tool in nutritional assessment, its clinical utility is often limited by high operator dependency. Artificial Intelligence (AI) overcomes this barrier by automatically recognizing complex anatomical patterns and providing precise, reproducible quantitative data. In the specific context of nutritional assessment, AI-driven analysis significantly reduces inter-operator variability and enhances the evaluation of muscle quality, offering a crucial framework for standardizing measurements (12). Despite the advantages of speed and standardization, the validation of these ultrasound-based AI models currently relies on comparative studies cross-referencing multiple established diagnostic modalities (13, 14). Beyond technical validation, it is critical to determine the prognostic utility of advanced AI-derived markers. Specifically, radiomic features of muscle quality hold significant promise; by characterizing tissue microarchitecture, these radiomic markers can detect subclinical pathophysiological changes well before overt clinical deterioration manifests (15).

The purpose of this study is to evaluate the prognostic influence of classic parameters determined by automatic segmentation tools, as well as novel muscle quality biomarkers derived from these technologies. Accordingly, the primary objective of this study was to assess the prognostic impact of AI-assisted ultrasound markers of muscle quantity and quality in oncology patients, while simultaneously evaluating the overall diagnostic capacity of these AI-analyzed ultrasound variables.

## 2 Materials and methods

### 2.1 Study design

This prospective, open-label, longitudinal cohort study evaluates patients’ nutritional status through comprehensive nutritional assessment, including dietary intake, anthropometry, nutritional ultrasound, and muscle strength. Following ethical approval from the Valladolid Clinical Research Ethics Committee (PI 22–2,907 on 13 October 2022 to initial recruitment and evaluation and PI-25-536-C for AI image analysis, with date 10 September 2025) and the collection of written informed consent, extensive medical, demographic, and nutritional histories were recorded to establish baseline profiles for all participants.

A core component of the methodology involved analyzing ultrasound images from these patients, acquired by a single investigator, with an AI-assisted online body composition system (PIIXMEDTM, Dawako MedTech, Spain) in the context of malnutrition assessment. Finally, the collected data underwent a descriptive statistical analysis to assess the prevalence of malnutrition and compare the different morphofunctional techniques. This was followed by univariate and multivariate inferential analyses designed to evaluate the prognostic impact of the baseline AI-assisted muscle ultrasound values on patient outcomes.

### 2.2 Patient selection

Patients were recruited at the Clinical Nutrition clinic following referral from the Oncology department between July 2023 and December 2025, with follow-up continuing until June 2026. The inclusion criteria were patients aged 18 years or older at risk of malnutrition, or outpatients referred to the nutrition clinic due to high nutritional risk. The exclusion criteria included the presence of decompensated liver disease, chronic kidney disease (CKD) stage IV or higher, or failure to provide signed informed consent.

### 2.3 Study variables

#### 2.3.1 Clinical variables and outcomes

Age (years) and sex (male/female) were collected. Regarding the underlying pathology, tumor location was recorded by categorizing each cancer into a standardized site (esophagogastric, colorectal, hepatopancreatobiliary, urological, gynecological, hematological, head and neck, and others) and it was classified as digestive vs. non digestive location. Furthermore, the tumor stage was evaluated and the presence or absence of metastatic disease. Following the recommendations of the International Society of Geriatric Oncology (SIOG) for geriatric assessment, the threshold for defining a patient as geriatric was set at >70 years (16). As for the outcome variables, all-cause mortality was assessed, recording the exact date of death for those who died, while event-free patients were censored at the time of their last clinical evaluation.

#### 2.3.2 Anthropometry

Anthropometric assessment included the recording of usual and current body weight (kg), height (m), and body mass index (BMI, kg/m^2^). Additionally, the percentage of weight loss (%) was calculated, and both mid-upper arm circumference and calf circumference (cm) were measured.

#### 2.3.3 Muscle ultrasonography

- *Image acquisition:* Ultrasound assessment of the rectus femoris muscle in the dominant leg was performed using a 10 MHz multifrequency 7L4P linear probe in MSK mode (Mindray Z60, Madrid, Spain). Examinations were conducted with the patient in a supine position, placing the probe perpendicular to the muscle on the transverse plane, at the lower third of the distance between the iliac crest and the superior patellar border (17). All scans were obtained by trained personnel following a standardized protocol, applying uniform settings across patients (frequency 10 MHz, depth 4.5–4.7 cm; gain 43–45; frame rate 23 fps; dynamic range 155). Operators received specific training in muscle imaging, including probe placement, image optimization, and techniques to minimize tissue compression. Only minimal pressure was applied, sufficient for image clarity but without deforming subcutaneous tissue or muscle, although compression was not quantitatively monitored. For each participant, three transverse images and three longitudinal images of the rectus femoris were acquired. The highest-quality image, judged by clarity and anatomical definition, was selected for analysis. Images were stored in JPEG format, and although this involves lossy compression, care was taken to avoid loss of diagnostically relevant information.
- *Artificial-Intelligence Image Analysis:* Ultrasound images were processed using PIIXMED™ MSK (DAWAKO Medtech S.L., Valencia, Spain), a cloud-based image-analysis platform incorporating target-specific convolutional neural networks based on the U-Net architecture. Separate models are used according to the acquisition plane and anatomical target. For the present study, the relevant models delineated the rectus femoris muscle in transverse images and the anatomical boundaries required to quantify subcutaneous adipose tissue and muscle architecture in longitudinal images. The neural networks perform anatomical segmentation, whereas the subsequent calculation of thickness, cross-sectional area, pennation angle, echogenicity, and texture variables is performed through deterministic image-processing and radiomics procedures. The models operate in a locked inference configuration and do not undergo online or case-specific learning during clinical use.

The models were developed using de-identified grayscale B-mode ultrasound images paired with expert-generated segmentation masks. Associated metadata identified the acquisition plane, laterality, segmented anatomical structure, and target muscle. Because separate task-specific models were developed for different anatomical targets, no single pooled training-image count accurately represents the complete platform. Depending on the segmentation task, the development datasets contained 278, 520, or 720 annotated images. During historical model development, each dataset was divided into 80% training and 20% hold-out subsets, and this procedure was repeated five times after reordering the samples to assess performance stability across different partitions. Across the documented model-development evaluations, intersection over union ranged from 0.8150 to 0.9907, pixel accuracy from 0.9640 to 0.9957, mean accuracy from 0.8794 to 0.9950, and frequency-weighted intersection over union from 0.9409 to 0.9915. These values represent internal model-development evidence rather than independent external clinical validation.

The clinical agreement of the automated measurements with conventional ultrasound measurements was previously evaluated in an independent cohort of 100 adults with disease-related malnutrition. The intraclass correlation coefficients were 0.912 (95% CI: 0.872–0.940) for subcutaneous fat thickness, 0.960 (95% CI: 0.941–0.973) for rectus femoris muscle thickness, and 0.995 (95% CI: 0.993–0.997) for rectus femoris cross-sectional area, with Bland–Altman analyses showing no evidence of substantial systematic bias between methods (12). Concurrent clinical validity in an oncological population was subsequently examined by comparing AI-assisted rectus femoris ultrasound variables with computed-tomography measurements at the L3 level in 337 patients (14). The current cohort includes a sub-sample of patients from this previously reported study by our group (14), exclusively selecting those who were undergoing active oncological treatment. Furthermore, this initial sample was expanded with an additional year of prospective patient recruitment to achieve the final cohort size.

Within the AI-segmented rectus femoris region of interest, muscle quality was characterized using deterministic image-processing procedures. Mean echo intensity was calculated on an 8-bit grayscale ranging from 0 to 255. In addition, a Multi-Otsu histogram-thresholding algorithm divided the pixels into three echogenicity classes by minimizing within-class variance and maximizing between-class variance: low echogenicity, reported as the Muscle Index (Mi); medium echogenicity, reported as the Fat Index (FATi); and high echogenicity, reported as the non-muscle/non-fat index (NMNFi). These indices were expressed as percentages of the segmented region of interest (18). Myosteatosis was operationally characterized by a lower Mi or higher FATi, using the previously established thresholds of 47 and 41%, respectively (14).

#### 2.3.4 Muscle functionality

Handgrip strength, used as a measure of muscle functionality, was assessed with a JAMAR® dynamometer (Basel, Switzerland). The measurement was taken with patients in a seated position, keeping the elbow of their dominant arm flexed at a right angle.

#### 2.3.5 Nutritional diagnosis

Patient nutritional and functional status was categorized as follows:

- *Sarcopenia:* Diagnosed according to European Working Group on Sarcopenia in Older People (EWGSOP2) criteria, which require the presence of low muscle strength combined with low muscle mass. Low muscle strength was defined as <27 kg for men and < 16 kg for women (19). For this study, muscle quantity was evaluated by measuring the thickness of the rectus femoris using ultrasound, applying the cutoff points established by López-Gómez et al. (validated against CT scans): <1.180 cm for men and <0.868 cm for women (14). Although the definition of sarcopenia includes low muscle quality, EWGSOP2 does not establish a specific range or measurement tool for it. Therefore, we did not use muscle quality as a diagnostic criterion for sarcopenia. Sarcopenia was defined according to the EWGSOP2 revised consensus. Specifically, “probable sarcopenia” was identified by the presence of low muscle strength alone, whereas “confirmed sarcopenia” was diagnosed when low muscle strength was accompanied by low muscle quantity (19).
- *Malnutrition:* Diagnosed using the GLIM criteria, requiring the presence of at least one phenotypic criterion (unintentional weight loss, low BMI, or reduced muscle mass, the latter also determined via the rectus femoris thickness cutoffs) alongside at least one etiologic criterion (reduced dietary intake/assimilation or disease-related inflammation) (20).

### 2.4 Statistical analysis

The database was registered with the National Data Protection Agency. Data were stored in a database using the SPSS 29.0.1.1 statistical software package (SPSS Inc., Chicago, IL, United States) under an official license from University of Valladolid.

The normality was assessed using the Kolmogorov–Smirnov test, and these are expressed as mean ± standard deviation for parametric variables and median (p25–p75) for non-parametric variables. Parametric variables were analyzed using paired and unpaired Student’s *t*-tests, while non-parametric variables were evaluated using the Friedman, Wilcoxon, Kruskal-Wallis, and Mann–Whitney U tests. Qualitative variables are expressed as percentages (%) and were analyzed using the Chi-square test (with Fisher’s exact test and Yates’ correction applied when necessary).

The primary outcome of this study was all-cause mortality. Survival time was calculated from the date of nutritional assessment to the date of death or last follow-up at the end of study by June 2026. Patients who did not experience the event by the end of the study period or who were lost to follow-up were censored at the date of their last known contact. The median follow-up duration, censoring information, and completeness of follow-up were systematically recorded. Missing data were handled using complete case analysis (listwise deletion), excluding patients with missing covariate data from the multivariate models. Prognostic analysis was conducted in two phases. Initially, univariate survival analysis was conducted using the log-rank test, and the resulting survival probabilities were illustrated using Kaplan–Meier curves. Subsequently, given the time-to-event nature of the data, Cox proportional hazards regression models were used. Covariate selection for inclusion in the multivariate models was not automated but prespecified based on clinical rationale and existing literature, categorizing them into muscle quantity and quality parameters.

To ensure methodological robustness and transparency, collinearity diagnostics were performed prior to modeling by calculating the Variance Inflation Factor (VIF). Specifically, a VIF greater than 5 was observed between rectus femoris thickness (RFT) and rectus femoris area (RFA), indicating substantial collinearity. For this reason, to prevent statistical distortion, these quantity variables were separated into different analytical models. Meanwhile, muscle quality variables [pennation angle, muscle index (Mi)], and adipose tissue index (FATi) were evaluated in independent models. Mi and FATi were presented as dichotomous variables based on the cutoff points established by López-Gómez et al. compared to CT scans (14).

All multivariate Cox models were adjusted for key covariates identified as biologically relevant confounders: age and sex (due to their physiological impact on muscle mass and quality), as well as oncological stage (metastatic and no metastasis) and tumor location (digestive vs. non-digestive), given the severe influence of nutritional status in metastatic or digestive tract cancers. Model fit and calibration were assessed using the likelihood ratio test (Omnibus test), and the proportional hazards assumption was verified.

Finally, to strengthen clinical applicability and evaluate discriminative performance, Receiver Operating Characteristic (ROC) curve analyses were conducted. The Area Under the Curve (AUC), sensitivity, specificity, and positive and negative predictive values (PPV and NPV) were calculated. Cox results are presented as hazard ratios (HRs) with their 95% confidence intervals (CIs). A two-sided *p*-value of <0.05 was considered statistically significant.

## 3 Results

### 3.1 Sample description

A total of 389 patients were analyzed, 58.9% of whom were men. The mean age was 70.62 ± 10.81 years, with 206 patients (53.7%) aged 70 years or older, who were consequently classified as geriatric oncology patients. Median (p25–p75) follow-up of the patients was 481 (264–879.5) days. 103 patients died (26.5%) and median follow-up of these patients was 173 (87–340.75) days. Patients who survived had a median follow-up of 608 (352–1,041) days (50.67 (29.33–86.75) months). Between patients who survived 21.7% had one-year follow up; 30.2% patients had 2 years follow-up; 21.2% had 3 years follow up; 13.8% patients had 4 years follow-up; and 13.2% patients had 5 years follow-up.

Overall, patients showed a heterogeneous oncologic profile (Table 1). Esophagogastric cancer was the most frequent primary tumor, and stage IV disease represented the largest proportion of cases. Surgical management of the primary tumor was the most common therapeutic approach (Table 1). In addition, a relevant subset received multimodal treatment, including combined chemotherapy and radiotherapy (97 patients; 24.9%) and chemotherapy with immunotherapy (79 patients; 20.3%).

**Table 1:** Differences in comprehensive nutritional assessment according to mortality, stratified by sex.

Variables	Total (*n* = 389)	Men (*n* = 229)	Women (*n* = 160)	*p*-value
Age (years)	70.62 ± 10.81	70.45 ± 10.29	70.87 ± 11.53	0.71
Anthropometry				
WL (%)	10.81 ± 9.14	10.74 ± 8.48	10.92 ± 10.05	0.85
BMI (kg/m^2^)	24.33 ± 4.35	24.97 ± 4.22	23.40 ± 4.39	<0.01
Arm circumference (cm)	25.66 ± 3.49	26.29 ± 3.31	24.75 ± 3.56	<0.01
Calf circumference (cm)	33.17 ± 3.77	33.86 ± 3.81	32.19 ± 3.51	<0.01
Rectus femoris ultrasonography (artificial intelligence assisted)				
SAT (cm)	0.73 ± 0.46	0.54 ± 0.36	1.01 ± 0.45	<0.01
RFT (cm)	0.99 ± 0.26	1.06 ± 0.25	0.87 ± 0.22	<0.01
RFA (cm^2^)	3.19 ± 1.04	3.57 ± 1.00	2.66 ± 0.85	<0.01
Echointensity (mean gray value 0–255)	92.21 ± 25.54	82.93 ± 23.56	105.45 ± 22.26	<0.01
Mi (%)	44.05 ± 7.04	45.72 ± 7.07	41.66 ± 6.27	<0.01
FATi (%)	39.81 ± 5.03	39.42 ± 5.02	40.38 ± 4.99	0.06
NMNFi (%)	16.14 ± 4.49	14.86 ± 4.19	17.96 ± 4.30	<0.01
AreaMi (cm^2^)	1.42 ± 0.54	1.63 ± 0.52	1.12 ± 0.42	<0.01
Area FATi (cm^2^)	1.27 ± 0.45	1.41 ± 0.46	1.07 ± 0.37	<0.01
Area NMNFi (cm^2^)	0.50 ± 0.19	0.53 ± 0.19	0.47 ± 0.17	<0.01
Pennation Angle (°)	6.14 ± 3.01	6.34 ± 3.05	5.85 ± 2.95	0.12
Muscle function				
Handgrip strength (kg)	25.31 (8.62)	29.84 (7.45)	18.58 (5.15)	<0.01
Nutritional diagnosis				
Malnutrition (%)	293 (75.3%)	173 (75.5%)	120 (75%)	0.91
Severe malnutrition (%)	171 (44%)	99 (43.2%)	72 (45%)	0.73
Probable sarcopenia (low muscle strength) (%)	111 (28.5%)	63 (27.5%)	48 (30%)	0.59
Low muscle mass (%)	204 (52.4%)	123 (53.7%)	81 (50.6%)	0.55
Confirmed sarcopenia (low muscle strength + low muscle mass) (%)	73 (18.8%)	44 (19.2%)	29 (18.1%)	0.79
Cancer location				
Esophagogastric	124 (32%)	77 (33.6%)	47 (29.4%)	0.01
Colorectal	100 (25.8%)	68 (29.7%)	32 (20%)	
Hepatobiliary-pancreatic	55 (14.2%)	24 (10.5%)	31 (19.4%)	
Head and neck	41 (10.5%)	33 (14.4%)	8 (5%)	
Urologic	22 (5.7)	19 (8.3%)	3 (1.9%)	
Lung	19 (4.9)	5 (2.2%)	14 (8.8%)	
Gynecologic	17 (4.4)	–	17 (10.6%)	
Hematological	5 (1.3)	1 (0.4%)	4 (2.5%)	
Other	5 (1.3)	2 (0.9%)	4 (2.5%)	
Cancer stage				
I	58 (14.9%)	26 (11.3%)	32 (20%)	0.01
II	62 (15.9%)	37 (16.1%)	25 (15.6%)	
III	134 (34.4%)	81 (35.4%)	53 (33.1%)	
IV	135 (34.7%)	82 (35.8%)	53 (33.1%)	
Cancer treatment				
Surgery	285 (73.33%)	176 (76.8%)	109 (68.1%)	0.12
Chemotherapy	279 (71.7%)	164 (71.6%)	115 (71.9%)	0.95
Radiotherapy	106 (27.2%)	72 (31.4%)	34 (21.3%)	0.03
Immunotherapy	85 (21.9%)	51 (22.3%)	34 (21.3%)	0.81

%WL, percentage weight loss; BMI, Body Mass Index; SAT, Subcutaneous Adipose Tissue; RFT, Rectus Femoris Thickness; RFA, Rectus Femoris Area; Mi, Low echogenicity percentage (muscle index); FATi, Medium echogenicity percentage (fat index); NMNFi, High echogenicity percentage (no muscle no fat index). Data are represented as Mean ± Standard Deviation for continuous variables and as *n* (% patients) in categorical variables. *p*-values represent baseline statistical comparisons between male and female subgroups (unpaired Student’s *t*-test or Mann–Whitney U test for continuous variables; omnibus Chi-square test or Fisher’s exact test for categorical variables evaluated as a single block to avoid Type I errors).

Regarding nutritional diagnosis, 75.3% of patients met GLIM criteria for malnutrition, and 44% had severe malnutrition. Confirmed sarcopenia was present in 18.8% of patients (28.5% had low handgrip strength and 52.4% had low muscle mass).

The comprehensive nutritional assessment stratified by sex is presented in Table 1 showing higher muscle mass, fat mass, and strength values in men.

When applying the cut-off points proposed by López-Gómez et al. for muscle quality (Mi and FATi) in sarcopenia, we observed that 66.6% of the sample had a value below 47% for Mi, and 40.9% had a value above 41% for FATi. A total of 28.5% of patients presented both an increased FATi percentage and a decreased Mi percentage, using the cut-off points established by López-Gómez et al.

### 3.2 Differences in body composition according to age

Out of the total sample of 389 patients, 206 (53.7%) were aged 70 years or older and categorized into the geriatric oncology cohort according to SIOG guidelines. When comparing baseline morphofunctional parameters between age groups, patients age ≥70 years presented significantly lower rectus femoris muscle quantity metrics, including reduced rectus femoris thickness (RFT) and cross-sectional area (RFA), alongside lower handgrip strength compared to non-geriatric patients (<70 years). However, no significant differences were observed in the categorical diagnosis of malnutrition (GLIM criteria) or confirmed sarcopenia (EWGSOP2 criteria) between age brackets (Table 2).

**Table 2:** Total and sex-related differences in comprehensive nutritional assessment stratified by age (<70 vs. >70 years).

Variables	Total (*n* = 389)			Men (*n* = 229)			Women (*n* = 160)			Men vs. Women	
	≥70 years (*n* = 209)	<70 years (*n* = 180)	*p*-value	≥70 years (*n* = 124)	<70 years (*n* = 105)	*p*-value	≥70 years (*n* = 85)	<70 years (*n* = 75)	*p*-value	*p*-value (≥70 years)	*p*-value (<70 years)
Age (years)	78.59 ± 5.07	61.37 ± 7.95	<0.01	77.9 ± 4.68	61.62 ± 7.81	<0.01	79.55 ± 5.47	61.03 ± 8.17	<0.01	0.02	0.62
Anthropometry											
WL (%)	11.15 ± 8.68	10.43 ± 9.66	0.45	11.31 ± 7.52	10.06 ± 9.49	0.28	10.91 ± 10.22	10.94 ± 9.93	0.98	0.75	0.56
BMI (kg/m^2^)	24.66 ± 3.92	23.94 ± 4.79	0.11	24.79 ± 3.56	25.18 ± 4.89	0.50	24.45 ± 4.41	22.21 ± 4.08	<0.01	0.53	<0.01
Arm circumference (cm)	25.36 ± 3.17	26.01 ± 3.81	0.07	25.66 ± 2.85	27.04 ± 3.66	<0.01	24.92 ± 3.56	24.58 ± 3.57	0.55	0.09	<0.01
Calf circumference (cm)	32.75 ± 3.42	33.67 ± 4.10	0.02	33.08 ± 3.37	34.79 ± 4.09	<0.01	32.26 ± 3.45	24.58 ± 3.57	0.81	0.08	<0.01
Rectus femoris ultrasonography (artificial intelligence assisted)											
SAT (cm)	0.68 ± 0.42	0.79 ± 0.49	0.03	0.51 ± 0.31	0.58 ± 0.41	0.10	0.95 ± 0.43	1.07 ± 0.46	0.08	<0.01	<0.01
RFT (cm)	0.95 ± 0.24	1.03 ± 0.28	<0.01	1.01 ± 0.24	1.12 ± 0.26	<0.01	0.86 ± 0.20	0.89 ± 0.24	0.27	<0.01	<0.01
RFA (cm^2^)	3.07 ± 0.98	3.34 ± 1.09	0.01	3.39 ± 0.97	3.77 ± 1.01	<0.01	2.59 ± 0.78	2.74 ± 0.92	0.25	<0.01	<0.01
Echointensity (mean gray value 0–255)	94.11 ± 24.736	89.96 ± 26.36	0.11	85.12 ± 22.70	80.30 ± 24.41	0.13	107.23 ± 21.59	103.41 ± 22.97	0.28	<0.01	<0.01
Mi (%)	43.71 ± 7.24	44.45 ± 6.78	0.29	45.47 ± 7.42	46.03 ± 6.65	0.56	41.13 ± 6.15	42.27 ± 6.38	0.26	<0.01	<0.01
FATi (%)	39.86 ± 5.02	39.76 ± 5.06	0.86	39.26 ± 5.15	39.60 ± 4.89	0.61	40.73 ± 4.72	39.98 ± 5.31	0.35	0.04	0.62
NMNFi (%)	16.44 ± 4.56	15.78 ± 4.41	0.15	15.27 ± 4.41	14.37 ± 3.86	0.11	18.14 ± 4.24	17.75 ± 4.39	0.57	<0.01	0.01
AreaMi (cm^2^)	1.35 ± 0.51	1.50 ± 0.57	<0.01	1.54 ± 0.48	1.75 ± 0.55	<0.01	1.08 ± 0.40	1.17 ± 0.43	0.18	<0.01	<0.01
Area FATi (cm^2^)	1.22 ± 0.43	1.33 ± 0.47	0.02	1.34 ± 0.45	1.49 ± 0.44	0.01	1.05 ± 0.33	1.09 ± 0.41	0.42	<0.01	<0.01
Area NMNFi (cm^2^)	0.49 ± 1.92	0.51 ± 0.18	0.46	0.52 ± 0.21	0.53 ± 0.17	0.63	0.46 ± 0.15	0.48 ± 0.19	0.51	0.03	0.06
Pennation angle (°)	5.88 ± 2.93	6.45 ± 3.09	0.64	6.09 ± 2.88	6.65 ± 3.23	0.16	5.58 ± 2.99	6.17 ± 2.88	0.21	0.22	0.31
Muscle function											
Handgrip strength (kg)	23.3 (7.59)	27.85 (9.18)	<0.01	27.52 (5.98)	32.79 (8.09)	<0.01	16.97 (4.87)	20.58 (4.81)	<0.01	<0.01	<0.01
Nutritional diagnosis											
*n* (%)	>70 years (*n* = 209)	<70 years (*n* = 180)	*p*-value	>70 years (*n* = 124)	<70 years (*n* = 105)	*p*-value	>70 years (*n* = 85)	<70 years (*n* = 75)	*p*-value		
Malnutrition *n* (%)	158 (75.6)	135 (75)	0.89	98 (79)	75 (71.4)	0.18	60 (70.6)	60 (80)	0.17	0.16	0.19
Severe malnutrition *n* (%)	89 (42.6)	82 (45.6)	0.56	54 (43.5)	45 (42.9)	0.92	35 (41.2)	37 (49.3)	0.31	0.73	0.39
Probable sarcopenia (low muscle strength) *n* (%)	57 (27.3)	54 (30)	0.55	35 (28.2)	28 (26.7)	0.79	22 (25.9)	26 (34.7)	0.23	0.71	0.25
Low muscle mass *n* (%)	113 (54.1)	91 (50.6)	0.49	70 (56.5)	53 (50.5)	0.37	43 (50.6)	38 (50.7)	0.99	0.40	0.98
Confirmed sarcopenia (low muscle strength + low muscle mass) *n* (%)	37 (17.7)	36 (20)	0.56	22 (17.7)	22 (21)	0.33	15 (17.6)	14 (18.7)	0.87	0.99	0.70

%WL, percentage weight loss; BMI, Body Mass Index; SAT, Subcutaneous Adipose Tissue; RFT, Rectus Femoris Thickness; RFA, Rectus Femoris Area; Mi, Low echogenicity percentage (muscle index); FATi, Medium echogenicity percentage (fat index); NMNFi, High echogenicity percentage (no muscle no fat index). Data are represented as Mean ± Standard Deviation in continuous variables and as *n* (% patients) in categorical variables. *P*-values in columns represent statistical comparisons between age brackets (<70 years vs. ≥70 years) within the overall cohort and within each sex subgroup (unpaired Student’s *t*-test or Mann–Whitney U test for continuous variables; omnibus Chi-square test or Fisher’s exact test for categorical variables).

Furthermore, when stratifying the analysis by sex, prominent morphological and functional differences emerged, particularly within the geriatric cohort (>70 years). Older men demonstrated significantly higher rectus femoris thickness and cross-sectional area, as well as greater handgrip strength compared to older women. Conversely, women aged >70 years exhibited significantly higher subcutaneous adipose tissue and echointensity, suggesting differences in muscle quality and fat infiltration. Despite these marked sex-related disparities in muscle architecture and strength, the prevalence rates for malnutrition, low muscle mass, and confirmed sarcopenia showed no statistically significant differences between men and women in either age bracket (Table 2).

### 3.3 Survival analysis

Kaplan–Meier survival analysis and Log-rank tests were performed to evaluate differences in cumulative survival. Patients with low muscle mass evaluated by Rectus Femoris Thickness showed an estimated survival time of 41.06 months (95%CI: 37.47–44.64) compared to 42.10 months (95% CI: 39.18–44.19) in those without low muscle mass, with no significant differences in survival (Log-rank *p* = 0.119) (Figure 1). However, a significantly lower survival time was observed in patients with a Mi Area <1.36 cm^2^ (38.66 months; 95% CI: 34.99–42.33) compared to those with an area > 1.36 cm^2^ (44.72 months; 95% CI: 41.36–48.07) (Log-rank *p* = 0.014) (Figure 1).

**Figure 1:**
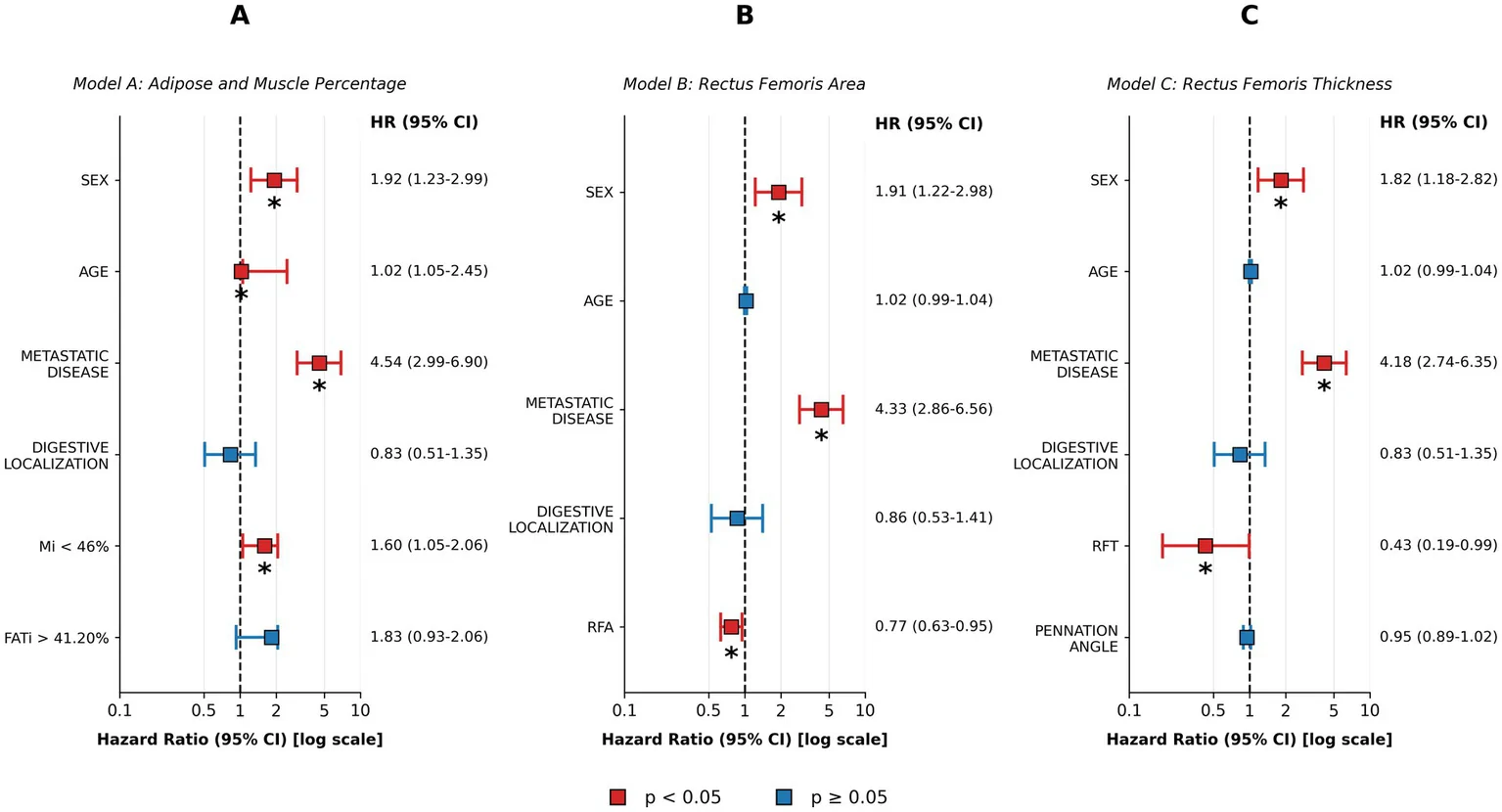
Forest plots showing adjusted hazard ratios (HR) and 95% confidence intervals (CI) for mortality from three Cox proportional hazards regression models, each adjusted for a different muscle parameter: muscle quality **(A)**, muscle mass **(B)**, and contractility **(C)**, together with clinical covariates (sex, age, metastatic disease, digestive localization). Squares represent the point estimate of the HR; horizontal lines represent the 95% CI. The dashed vertical line indicates HR=1 (no association with mortality). Variables with a statistically significant association (*p*<0.05) are shown in red and marked with an asterisk (*). To the right of each model, the FR and its 95% CI (in parentheses) are shown. Qualitative variables: Sex (1: Men; 0: Women); Metastatic disease (1: Yes; 0:No); Digestive localization (1:Yes; 2:No). HR, hazard ratio; RFA, rectus femoris area; FATi, medium echogenicity ROI percentage (fat index); Mi, low echogenicity ROI percentage (muscle index); RFT, rectus femoris thickness. This figure was created with the assistance of Claude (Anthropic; Opus 4.8), used solely for the graphical development of the figure and not for data analysis. The input prompt used is provided in Supplementary File 1.

Regarding tissue indices, patients with a Mi < 46% had a significantly lower estimated survival (38.74 months; 95%CI: 35.54–41.94) than those with an index >46% (46.23 months; 95% CI: 42.51–49.96) (Log-rank *p* = 0.019) (Figure 2). Conversely, the Fat Index (FATi) classification did not show statistically significant differences in survival (Log-rank *p* = 0.28), presenting an estimated survival of 39.51 months (95% CI: 35.60–43.43) for FATi >41% and 43.04 months (95% CI: 39.87–46.21) for FATi <41% (Figure 2). Finally, in patients with myosteatosis (defined as high FATi and low Mi), the Log-rank test showed a significantly lower survival (Chi-square 4.39; *p* = 0.04).

**Figure 2:**
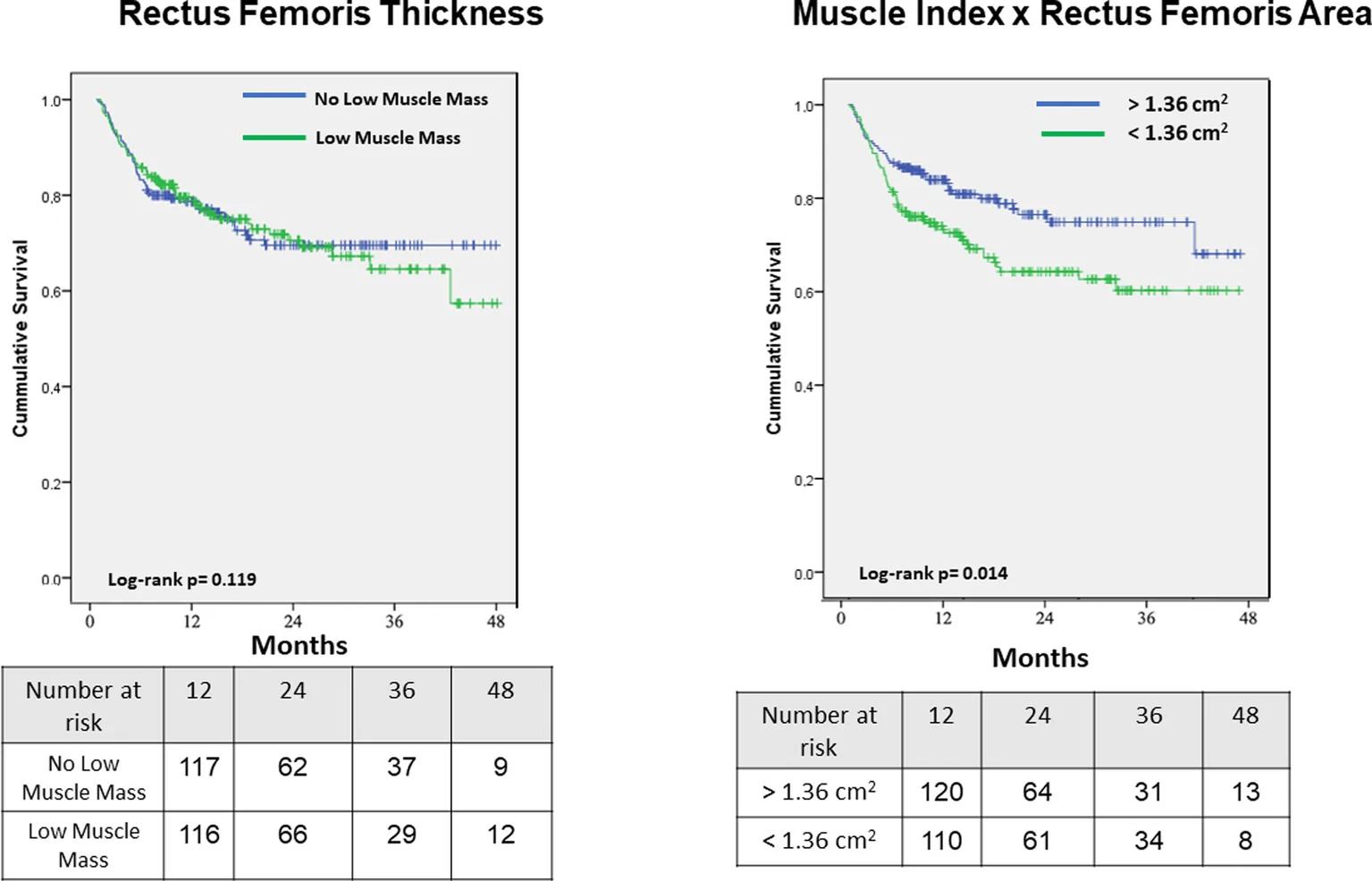
Kaplan -Meier curves comparing low muscle mass using rectus femoris thickness cut-off values, and the relationship between rectus femoris area and the percentage of low-echogenicity areas (MiArea).

### 3.4 Multivariate analysis

A Cox Regression was performed to evaluate mortality risk in relation to the main variables in oncology patients. Several models were developed and adjusted for sex, age, digestive cancer site, and metastatic disease. In the model assessing the relationship with muscle quantity, an increase in muscle area was observed to be a protective factor against mortality (Figure 3).

**Figure 3:**
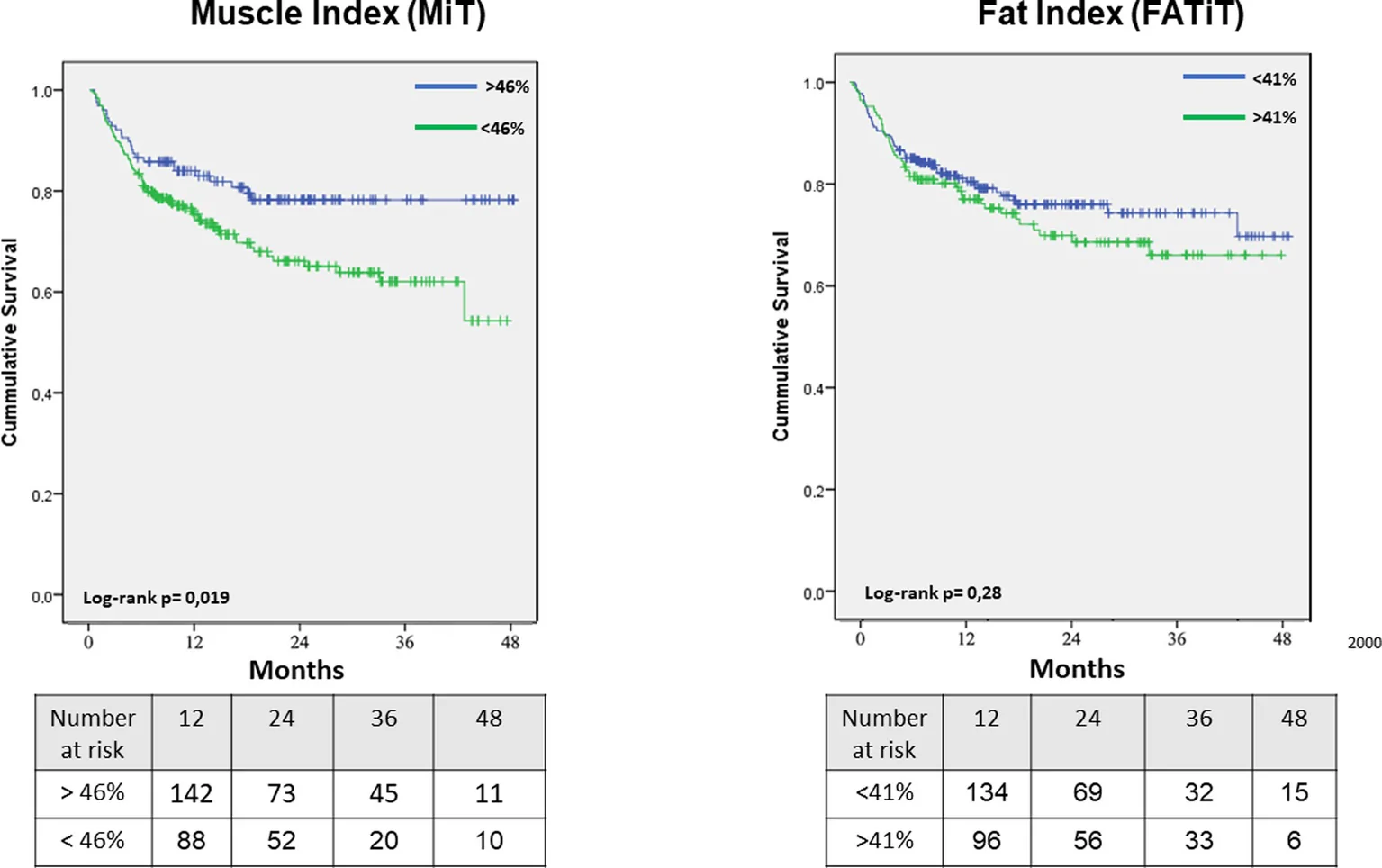
Kaplan -Meier curves comparing the percentage of muscle in the rectus femoris region of interest (Mi) and the percentage of fat (FATi).

In the muscle quality model, the cut-off points from the validation study of the technique by López-Gómez et al. (14) were applied. Being below the cut-off point for the percentage of muscle mass (Mi) in the ROI was associated with an increased risk of mortality, whereas this was not observed for the percentage of fat mass (FATi) (Figure 3). Defining myosteatosis as the coexistence of elevated FATi and reduced Mi, and entering this variable into another model, revealed an independent association with mortality (HR = 2.35, 95%CI: 1.19–4.62; *p* = 0.01) after adjustment for metastatic disease, sex, age, and digestive tumor site. Global model fit assessed via the omnibus test yielded statistically significant results for all evaluated configurations, with a chi-square of 84.89 (*p* < 0.01) for Model A, 81.66 (*p* < 0.01) for Model B, and 80.61 (*p* < 0.01) for Model C (Figure 3).

In the model evaluating contractile potential, rectus femoris thickness was found to have a protective effect on mortality (Figure 1). The Echo intensity model adjusted for the same variables, did not show significance in the relationship (HR = 1.09 (95%CI: 0.61–1.93); *p*-value = 0.77). Similarly, the model evaluating low muscle, adjusted for the same covariates, did not reach statistical significance (HR:1.020 (CI95%:0.69–1.51); *p*-value 0.92).

### 3.5 Receiver operating characteristic analysis

The diagnostic performance and predictive values of the AI-assisted rectus femoris ultrasound parameters for mortality risk are summarized in Table 3 and illustrated in Figure 3. Among all evaluated metrics, muscle Echointensity demonstrated the highest area under the curve (AUC) for predicting mortality. Similarly, RFA, RFT and MiArea yielded significant AUC values of 0.59, displaying distinct performance profiles. The analysis revealed divergent predictive profiles among the assessed variables. RFA demonstrated a highly specific prognostic capacity (91.2%) and yielded the highest Positive Predictive Value (PPV) at 51.2% although its sensitivity was remarkably low (25.7%). Conversely, RFT, Mi, MiArea and FATiArea exhibited a more balanced diagnostic performance. These parameters showed sensitivities and specificities ranging between 50 and 62%, which translated into robust Negative Predictive Values (NPV) clustering around 78 to 80.2% making them more suitable for ruling out mortality risk, albeit with lower PPVs (32.4 to 35.3%). Notably the isolated FATi parameter displayed an extremely poor predictive capacity for this clinical endpoint, characterized by a very low sensitivity (3.2%). Additionally, the evaluation of muscle echointensity yielded an optimal cut-off point of 94.35, demonstrating a balanced diagnostic performance with a sensitivity of 53.52% and a specificity of 69.61%. This parameter achieved a low PPV but a robust negative predictive value, reflecting a higher specificity for mortality risk compared to other textural parameters and reinforcing its utility in risk stratification (Figure 4).

**Table 3:** Diagnostic performance and predictive values of artificial intelligence-assisted rectus femoris ultrasound parameters for mortality risk (prevalence of 26.5%), based on optimal Youden index cut-off points.

Variable (units)	Cut-off point	AUC (CI95%)	Sensitivity	Specificity	Positive predictive value	Negative predictive value
RFA (cm^2^)	4.036	0.59 (0.53–0.66)*	25.70%	91.20%	51.20%	77.30%
RFT (cm)	0.96	0.59 (0.53–0.65)*	57.75%	61.76%	35.36%	80.21%
Mi (%)	43%	0.54 (0.47–0.59)	55.99%	57.84%	32.38%	78.47%
FATi (%)	49.18%	0.43 (0.37–0.49)*	3.17%	98.04%	36.82%	73.74%
MiArea (cm^2^)	1.29	0.62 (0.55–0.68)*	58.80%	58.80%	33.97%	79.83%
FATiArea (cm^2^)	1.23	0.59 (0.52–0.65)*	51.40%	61.80%	32.67%	77.91%

RFT, Rectus Femoris Thickness; RFA, Rectus Femoris Area; Mi, Low echogenicity percentage (muscle index); FATi, Medium echogenicity percentage (fat index). * *p*-value <0.05.

**Figure 4:**
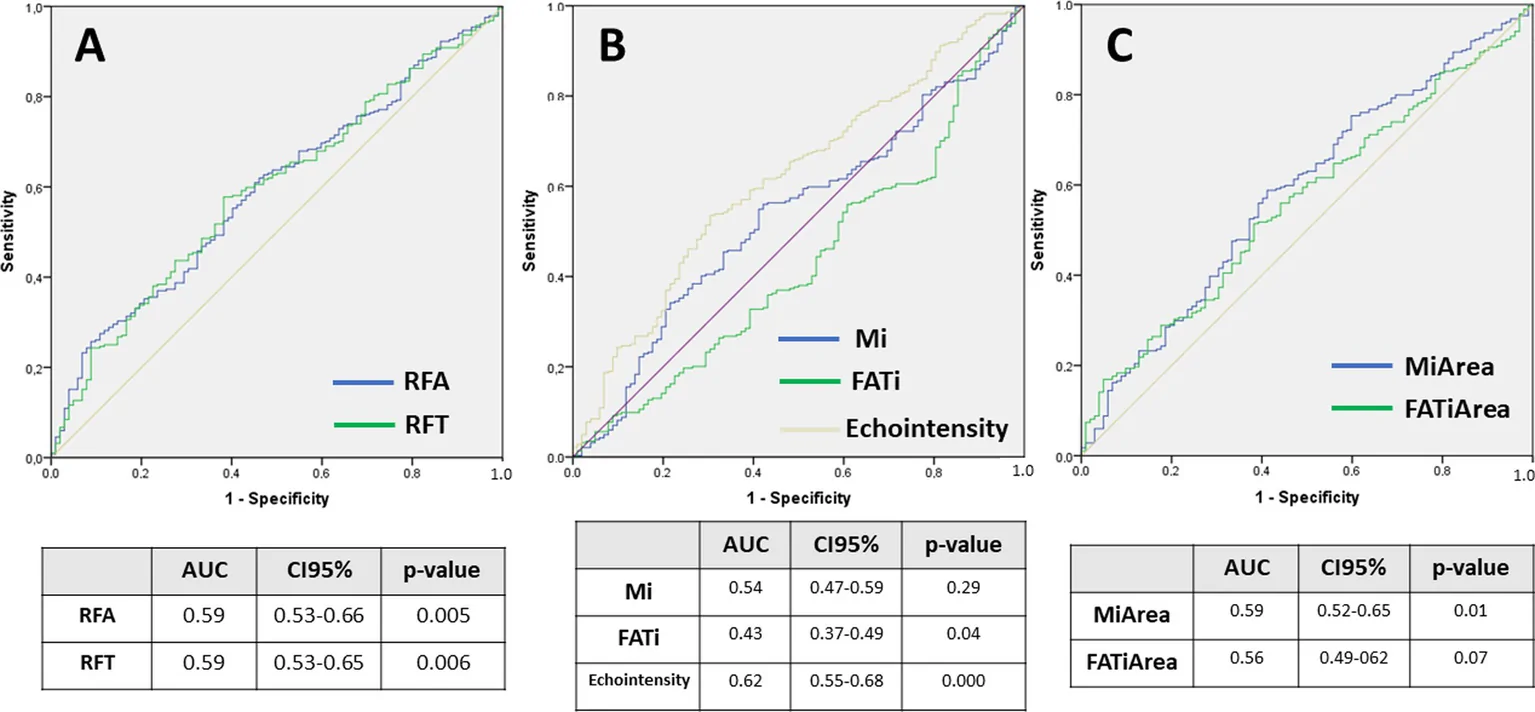
Receiver operating curves of quantity variables **(A)**, quality variables **(B)**, and combined quantity-quality variables **(C)**. RFT, Rectus Femoris Thickness; RFA, Rectus Femoris Area; Mi, Low echogenicity percentage (muscle index); FATi, Medium echogenicity percentage (fat index); MiArea, Muscle Index × Area.

## 4 Discussion

Patients with oncologic pathology have a high prevalence of malnutrition and sarcopenia. Artificial intelligence is a useful tool for measuring muscle mass and quality. Higher muscle mass measured by muscle ultrasound was associated with a more favorable prognosis against mortality in cancer patients, while patients with low muscle quality are associated with increased mortality. In fact, patients with better muscle mass and quality have a higher probability of survival.

Most patients in the sample were malnourished, and nearly half had severe malnutrition. The prevalence of malnutrition in the cancer populations using GLIM criteria ranges from approximately 29 to 47%, depending on the screening tool used (21). In our population the prevalence was higher because all patients evaluated in the study were already at risk of malnutrition; we did not calculate prevalence across all patients with oncological disease in the specialty setting. Sarcopenia was diagnosed in almost 20% of patients, and 28.5% had impaired muscle strength. Overall prevalence of sarcopenia in patients with cancer is approximately 33 to 42%, depending on diagnostic criteria, tumor type, and clinical setting (2, 22). This variability may be influenced by the cut-off points for muscle mass derived from muscle thickness measured by CT in our group (14). These cut-off points were chosen because ultrasonography was used to assess muscle mass in this study.

AI-assisted muscle ultrasound showed that 40.9% of patients had myosteatosis, defined as increased FATi (higher percentage of medium echogenicity within the ROI), and 66.6% had a reduced low-echogenicity (Mi) percentage in the ROI. 28.5% of patients presented both conditions (low Mi and high FATi) using the cut-off points established by López-Gómez et al. (14). A systematic review and meta-analysis by Aleixo et al. reported an overall mean prevalence of myosteatosis in cancer patients assessed by CT of approximately 48% (23). Myosteatosis represents a distinct muscle phenotype from sarcopenia, reflecting impaired muscle quality rather than reduced muscle quantity; it often appears early in the trajectory of muscle deterioration and has been associated with poorer prognosis (23, 24). Our sample shows the prevalence of increased intramuscular fat comparable to previously published series.

Our findings reveal a notable discrepancy between quantitative muscle assessments and standard categorical diagnoses. Although geriatric patients, particularly women demonstrate significant morphofunctional deterioration, such as reduced rectus femoris metrics, lower handgrip strength, and higher fat infiltration, these continuous declines do not translate into higher prevalence rates of malnutrition or confirmed sarcopenia in our cohort. This observed deterioration aligns with the physiological impact of aging, which inherently reduces muscle mass by approximately 0.5–1% annually after age 40 driven by hormonal declines (25); a process that cancer and its treatments can accelerate up to tenfold. While age-related primary sarcopenia often pre-exists the oncological diagnosis and overlaps with tumor cachexia, reaching prevalence rates of 60% in men and 56% in women aged ≥70 years with colorectal cancer, categorical criteria may fail to capture earlier structural shifts (26). As recent literature highlights, the prognostic relevance of these specific muscle parameters shifts with age: myosteatosis (fat infiltration) is heavily associated with survival in patients aged 51–74, whereas low muscle mass becomes the dominant prognostic marker in those ≥75 years (27).

The study underscores the profound impact of aging and sexual dimorphism on muscle architecture. The transition to the ≥ 70 years cohort is marked by significant muscle and strength loss that is heavily influenced by sex: older men retain greater muscle thickness and strength, while older women exhibit poorer muscle quality characterized by increased subcutaneous fat and echointensity. Surprisingly, these deep structural and functional disparities do not alter the overall diagnosis rates of malnutrition or sarcopenia, raising questions about whether current criteria adequately stratify risk among these vulnerable groups. There is evidence about differences in changes in muscle mass related to sex in cancer patients, with a sign that men with cancer experience more severe and more rapid deterioration of muscle mass and quality compared to women. Muscle mass during chemotherapy is approximately 1.6 times greater in men than women (28). Myosteatosis shows sex-specific prognostic impact, in advanced hospitalized cancer patients, female sex was independently associated with lower muscle radiodensity, though the prognostic impact of low radiodensity on survival was significant across both sexes (29). Importantly alterations in muscle quality often precede measurable changes in muscle mass and strength; because current diagnostic criteria for sarcopenia do not fully capture these early architectural and compositional shifts, conventional sarcopenia diagnosis remains challenging or undetermined in these phases (30).

Regarding overall survival analysis, our prospective findings demonstrate that AI-assisted muscle ultrasound metrics provide significant prognostic stratification when evaluated through adjusted Cox proportional hazards models. Increased rectus femoris cross-sectional area and muscle thickness were confirmed as independent protective factors against all-cause mortality. These prospective hazard estimates align with previous observations. Quadriceps rectus femoris thickness had demonstrated a relation with prognosis in patients with cancer in study Any Vida from García et al. (11). In patients with head and neck cancer lower values of rectus femoris area was the only independent prognosticator of mortality (HR = 4.42; *p* = 0.03) in the study of Galli et al. study (31). This parameter was related also to the development of postoperative complications in the same study (31). In our cohort, increased rectus femoris thickness and larger rectus femoris area were independently associated with reduced mortality. However, in time-to-event analysis, only a larger muscle area within the ROI was associated with prolonged survival; rectus femoris thickness showed no significant relationship with survival time. This divergence may be explained by the heterogeneity of the sample with respect to tumor localization and stage, which could differentially influence muscle morphology and its prognostic relevance.

Furthermore, qualitative tissue impairment evaluated via AI radiomic analysis demonstrated robust independent prognostic value. A reduced Muscle Index (Mi < 47%) was associated with a 60% increase in prospective mortality risk, while the composite presence of myosteatosis (low Mi and high FATi) conferred an HR of 2.35 (95%CI: 1.19–4.62; *p* = 0.01). Notably, while baseline continuous muscle mass and strength were lower in older adults (≥70 years), the independent association between qualitative ultrasound biomarkers (Mi and myosteatosis) and survival persisted after controlling for age in multivariate Cox models. This confirms that AI-derived qualitative muscle biomarkers capture specific cachectic microarchitectural degradation beyond physiological age-related sarcopenia. Echo intensity on ultrasound, a marker of intramuscular fat and fibrotic infiltration analogous to CT-derived myosteatosis, has been linked to adverse clinical phenotypes in cancer and surgical populations. In patients with lung cancer, rectus femoris mean gray value, indicating increased echogenicity, was higher than in benign controls and rose significantly with sarcopenia severity (32). In older patients undergoing surgery for gastrointestinal malignancy, greater echo intensity of the tibialis anterior and medial gastrocnemius was independently associated with frailty, and frailty predicted postoperative complications and poor outcomes (33). As systematic review of 37 studies found that, among studies measuring echo intensity, most reported significant associations with clinically relevant endpoints such as physiological status, quality of life, and functional status at discharge, supporting echo intensity as a meaningful prognostic biomarker (34). Therefore, a single echogenicity value should be complemented with histogram metrics, structural parameters, and functional or imaging correlates when possible. AI tools that segment muscle and analyze echogenicity histograms can enhance reproducibility but relying on a single echogenicity value risks misinterpretation because it conflates diverse tissue and technical influences; combining histogram-based metrics, structural measures, and functional data improves accuracy and clinical usefulness (13). In our cohort, muscle quality assessed by AI-based biomarkers using a multi-threshold algorithm, expressed as the percentage of the ROI occupied by low-echogenicity areas (Mi) and medium-echogenicity areas (FATi), was associated with mortality. A lower percentage of Mi was linked to a higher risk of death and shorter survival, whereas higher FATi alone was not significantly associated with mortality; however, the coexistence of low Mi and high FATi was associated with increased mortality. These results highlight the importance of evaluating echogenicity as the relative distribution of different echogenicity areas within the ROI rather than as a single summary value. Muscle quality assessment by echogenicity may be more clinically important than muscle mass measurement alone, as early evidence suggests muscle quality deteriorates before muscle mass and is independently associated with physical function and survival (34).

Regarding the diagnostic performance of AI-assisted nutritional ultrasound biomarkers for predicting mortality risk, our analysis demonstrates moderate discriminative power, with AUC values ranging between 0.54 and 0.62. Specifically, rectus femoris echointensity achieved the highest diagnostic accuracy for mortality alongside a high negative predictive value whereas rectus femoris area demonstrated elevated specifity and positive predictive value. Moreover, muscle quantity metrics such as rectus femoris thickness and muscle area index consistently yielded high NPVs, emphasizing their utility as initial bedside screening tools to rule out high mortality risk. These diagnostic capabilities align with findings from large-scale clinical cohorts, such as the nationwide DRECO study in hospitalized patients at nutritional risk, where rectus femoris ultrasound parameters exhibited robust NPVs ranging from 85 to 98% for identifying confirmed and severe sarcopenia (35). Similarly, in oncological outpatients, AI-assisted rectus femoris ultrasonography demonstrated comparable diagnostic accuracy (AUCs of 0.63–0.64 for sarcopenia and myosteatosis) when compared against gold-standard L3 computed tomography (CT), with quantitative ultrasound metrics (RFA, RFT) correlating with CT-derived skeletal muscle area and qualitative metrics (Mi, FATi) successfully detecting tissue-level myosteatosis (14). Furthermore, the construct validity of these tissue-organ level biomarkers is backed by population-based data from the VALORES study in 1,789 healthy adults, which established normative T-score cut-offs and confirmed moderate-to-strong convergent correlations between rectus femoris ultrasound measurements, bioelectrical impedance analysis-derived muscle mass, and handgrip strength (36). These results substantiate expert-endorsed body composition frameworks highlighting that nutritional ultrasound provides a non-invasive, objective, and reproducible tool for comprehensive nutritional assessment and prognostic risk stratification in clinical practice.

The present study has several strengths. It included a relatively large clinical cohort and used muscle ultrasound, a non-invasive, repeatable, and accessible bedside technique. The standardized acquisition protocol and AI-assisted post-acquisition analysis promoted consistency in image processing and enabled the simultaneous evaluation of muscle quantity and quality parameters.

Nevertheless, our study is subject to certain limitations. Its observational and single-center design precludes the establishment of definitive causal relationships. Additionally, the cohort is inherently heterogeneous due to the inclusion of various tumor origins grouped by broad anatomical locations, making tumor-specific stratification challenging and prone to statistical error due to small subgroup sample sizes. To partially mitigate this limitation and control for potential confounders such as variations in clinical management, treatment intent, and tumor stages, our multivariate analysis distinguished between advanced/metastatic stages, predominantly managed with systemic therapies or concurrent chemoradiotherapy, and non-metastatic stages, which are frequently managed primarily with curative surgery. Nonetheless, future research should focus on validating these ultrasound parameters in larger, homogeneous cohorts of specific oncological pathologies. While AI standardizes the analysis, the initial ultrasound image acquisition remains operator dependent. The muscle quality indices used in this study, including Mi and FATi, are surrogate imaging biomarkers rather than direct histological measurements. Although these indices have previously been evaluated against CT-derived measures of muscle quality, they remain indirect estimates and require further biological and clinical validation.

The AI-derived results may also be affected by biases originating from the model-development data, including the representation of age, sex, body composition, disease status, ultrasound equipment, acquisition settings, and anatomical variability, as well as variability in the expert-generated reference annotations. Although acquisition variability was reduced in the present study using a standardized protocol, a single ultrasound system, trained personnel, and uniform acquisition settings, these same conditions may restrict generalizability to other devices, operators, institutions, and patient populations. Furthermore, selection of the highest-quality image and storage in JPEG format may introduce selection and compression-related effects. The previously published studies support agreement with conventional ultrasound measurements and concurrent validity against computed tomography; however, prospective multicenter and device-diverse external validation, including pixel-level segmentation assessment and subgroup-specific performance analysis, remains warranted.

Based on our findings, several promising lines of future research emerge. First, targeted studies evaluating the specific impact of different tumor types on muscle architecture are warranted. Second, research with greater statistical power is needed to conclusively determine the prognostic role of intramuscular fat, muscle quality as represented by Mi, rectus femoris area, and pennation angle. Future research should focus also on validating these ultrasound parameters in larger, homogeneous cohorts of specific oncological pathologies. This approach will help overcome tumor-type confounding and establish more precise, pathology-tailored diagnostic cut-offs. Ultimately, larger multicenter studies incorporating external validation are highly recommended to confirm these findings and establish their routine clinical applicability.

## 5 Conclusion

Malnutrition and sarcopenia were highly prevalent in this cohort of patients with cancer. AI-assisted rectus femoris ultrasound variables reflecting muscle quantity and quality were associated with mortality. Greater rectus femoris area was associated with a more favorable prognosis, whereas and Mi below 47% was associated with shorter survival. These findings support the potential prognostic value of integrating standardized ultrasound-based body-composition assessment into comprehensive nutritional evaluation in oncology. Importantly, the early assessment of muscle quality allows for the detection of structural alterations before overt clinical deterioration occurs. This proactive monitoring provides a critical window for targeted interventions aimed at preserving muscle integrity and preventing severe disease-related complications.

## Data Availability

The datasets presented in this article are not readily available because ethics restriction. Requests to access the datasets should be directed to corresponding authors.

## Supplementary material

SUPPLEMENTARY FIGURE S1 Images from rectus femoris ultrasonography with artificial intelligence assistance (PIIXMEDTM). **(A)** Male 67 years pancreatic cancer, alive; **(B)** Male 68 years pancreatic cancer, deceased. Mi, Low echogenicity percentage (muscle index); FATi, Medium echogenicity percentage (fat index); NMNFi, High echogenicity percentage (no muscle no fat index).
