# Supplementary material for: Novel AI-derived muscle ultrasound biomarkers linking nutritional status and prognosis in cancer patients
Source: Front Nutr. 2026 Oct 14;13:1961328. doi: 10.3389/fnut.2026.1961328 (PMC13714818; doi:10.3389/fnut.2026.1961328)
Supplement: Supplementary file 1 [file Supplementary_file_1.pdf]

# SUPPLEMMENTARY MATERIAL S1. USE OF GENERATIVE AI IN THE PREPARATION OF FIGURE 1

1. **AI technology used:**

   AI technology used to assist in the creation of the forest-plot figure

   - Name: Claude
   - Version: 4.8
   - Model: Claude (Opus 4.8, Anthropic)
   - Source: Anthropic (https://www.anthropic.com)

   The figure (Cox regression forest plots, Models A-C) was created on September 17,2026. The tool was used solely to generate Python (matplotlib) code to visualize results previously calculated by the authors in SPSS.

2. **Input prompt**

   The Hazard ratios, 95% confidence intervals and p-values were provided to the tool by the authors, taken from the output their own statistical analysis as in the next table.

| MODEL A: Adipose and Muscle Percentage | | | |
|---|---|---|---|
| | **HR** | **CI 95%** | **p-value** |
| **SEX** | 1.92 | 1.23-2.99 | <0.01 |
| **AGE** | 1.02 | 1.05-2.45 | 0.02 |
| **DIGESTIVE LOCALIZATION** | 0.83 | 0.51-1.35 | 0.14 |
| **METASTATIC DISEASE** | 4.54 | 2.99-6.90 | <0.01 |
| **Mi < 46%** | 1.60 | 1.05-2.06 | <0.01 |
| **FATi > 41.20%** | 1.83 | 0.93-2.06 | 0.66 |
| **Model B: Rectus Femoris Area** | | | |
| **SEX** | 1.91 | 1.22-2.98 | <0.01 |
| **AGE** | 1.02 | 0.99-1.04 | 0.07 |
| **DIGESTIVE LOCALIZATION** | 0.86 | 0.53-1.41 | 0.19 |
| **METASTATIC DISEASE** | 4.33 | 2.86-6.56 | <0.01 |
| **RFA** | 0.77 | 0.63-0.95 | 0.02 |
| **Model C: Rectus Femoris Thickness** | | | |
| **SEX** | 1.82 | 1.18-2.82 | <0.01 |
| **AGE** | 1.02 | 0.99-1.04 | 0.07 |
| **DIGESTIVE LOCALIZATION** | 0.83 | 0.51-1.35 | 0.16 |
| **METASTATIC DISEASE** | 4.18 | 2.74-6.35 | <0.01 |
| **RFT** | 0.43 | 0.19-0.99 | 0.03 |
| **PENNATION ANGLE** | 0.92 | 0.83-1.02 | 0.13 |

Table: Cox regression forest plots, Models A-C

The prompt below specifies the data and the formatting of the figure:

Using Python (matplotlib), generate a Cox regression forest plot with 3 side-by-side panels (A, B, C) in a single figure of 1280x720 px (16:9, 96 dpi).
Save it as both TIFF and PNG.

DATA (variable, HR, 95% CI lower, 95% CI upper, p):

Panel A – "Model A: Adipose and Muscle Percentage"

| | | | | |
|---|---|---|---|---|
| SEX | 1.92 | 1.23 | 2.99 | <0.01 |
| AGE | 1.02 | 1.05 | 2.45 | 0.02 |
| METASTATIC DISEASE | 4.54 | 2.99 | 6.90 | <0.01 |
| DIGESTIVE LOCALIZATION | 0.83 | 0.51 | 1.35 | 0.14 |
| FATi > 41.20% | 1.83 | 0.93 | 2.06 | 0.66 |
| Mi < 46% | 1.60 | 1.05 | 2.06 | <0.01 |

Panel B – "Model B: Rectus Femoris Area"

| | | | | |
|---|---|---|---|---|
| SEX | 1.91 | 1.22 | 2.98 | <0.01 |
| AGE | 1.02 | 0.99 | 1.04 | 0.07 |
| METASTATIC DISEASE | 4.33 | 2.86 | 6.56 | <0.01 |
| DIGESTIVE LOCALIZATION | 0.86 | 0.53 | 1.41 | 0.19 |
| RFA | 0.77 | 0.63 | 0.95 | 0.02 |

Panel C – "Model C: Rectus Femoris Thickness"

| | | | | |
|---|---|---|---|---|
| SEX | 1.82 | 1.18 | 2.82 | <0.01 |
| AGE | 1.02 | 0.99 | 1.04 | 0.07 |
| METASTATIC DISEASE | 4.18 | 2.74 | 6.35 | <0.01 |
| DIGESTIVE LOCALIZATION | 0.83 | 0.51 | 1.35 | 0.16 |
| RFT | 0.43 | 0.19 | 0.99 | 0.03 |
| PENNATION ANGLE | 0.95 | 0.89 | 1.02 | 0.13 |

STYLE:

- Each panel: variables on the Y axis (uppercase labels, with a line break in "METASTATIC / DISEASE" and "DIGESTIVE / LOCALIZATION"), listed top to bottom in the order of the table. No top or right spines.
- X axis on a logarithmic scale from 0.1 to 10, with ticks at 0.1, 0.5, 1, 2, 5, 10. Axis label: "Hazard Ratio (95% CI) [log scale]" in bold.
- Dashed black vertical line at HR = 1 and a very faint vertical grid.
- Each variable: square marker with a black edge, and horizontal error bars (95% CI) with caps.
- Color: red (#d62728) if p < 0.05 and blue (#1f77b4) if p ≥ 0.05.
- Large black asterisk (*) just below the marker for variables with p < 0.05.
- Text column to the right of each panel showing "HR (LL-UL)" to 2 decimals (e.g., "1.92 (1.23-2.99)"), small font, aligned.
- Header "HR (95% CI)" above that column, small font, bold.
- Panel title: large bold letter ("A", "B", "C") with the model name below it in italics.
- Shared legend centered at the bottom: red square "p < 0.05" and blue square "p ≥ 0.05".
- Sans-serif font, white background, generous margins between panels.

3. **Outputs received**
   The tool returned the resulting figure in PNG and TIFF formats.

4. **Human oversight and responsibility**
   The statistical analysis was performed by the authors without the use of generative AI. The AI tool did not analyze, alter or interpret any data. The authors checked the values shown in the figure against the original regression tables and take full responsibility for the content of the figure and the manuscript.
